# Supplementary figures and images for: Sugars and Organic Acids in 25 Strawberry Cultivars: Qualitative and Quantitative Evaluation
Source: Plants (Basel). 2023 Jun 7;12(12):2238. doi: 10.3390/plants12122238 (PMC10305725; doi:10.3390/plants12122238)

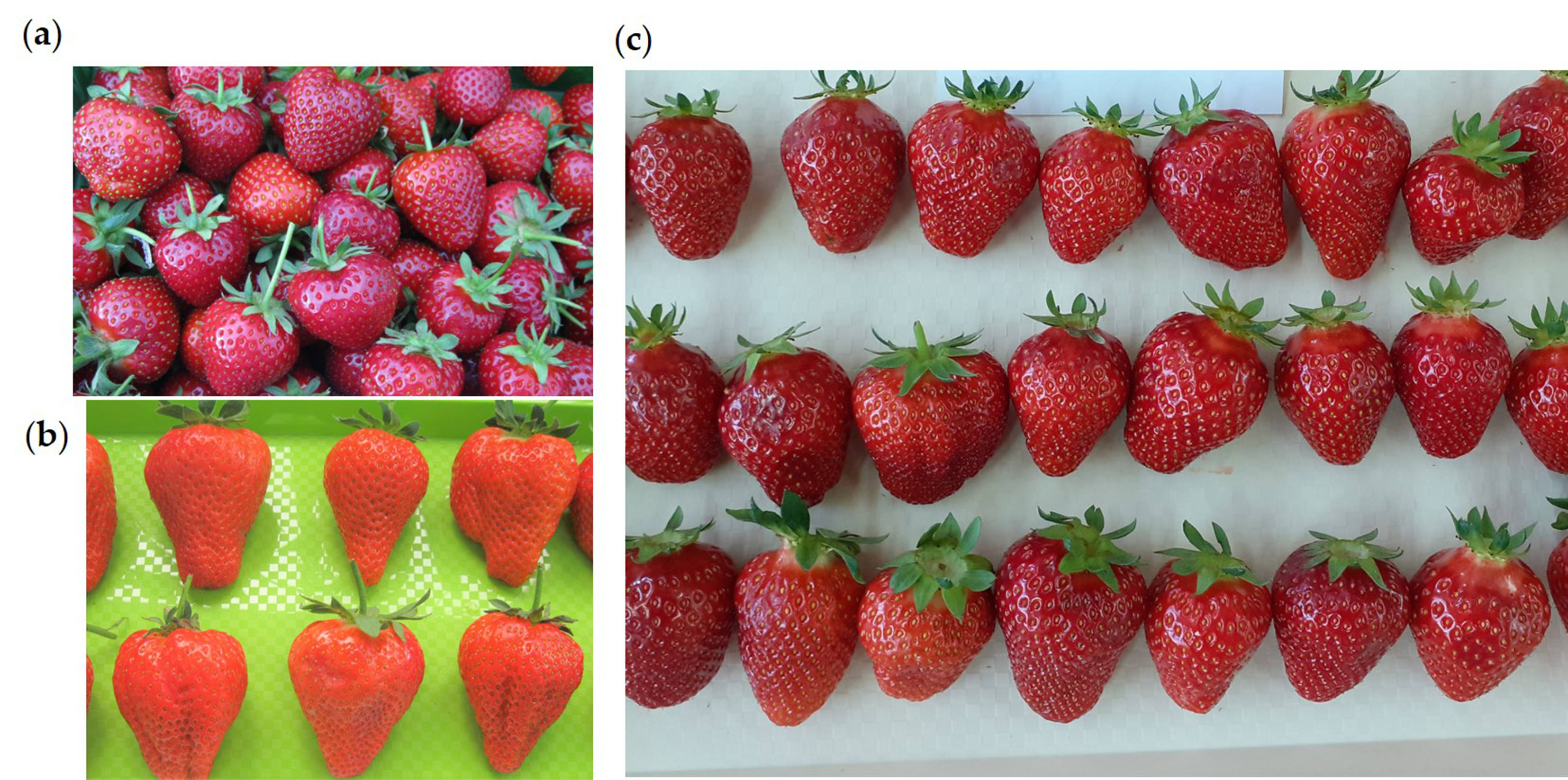

Supplement: Supplementary file 1 [file plants-12-02238-s001.zip › Figure S6.tif]

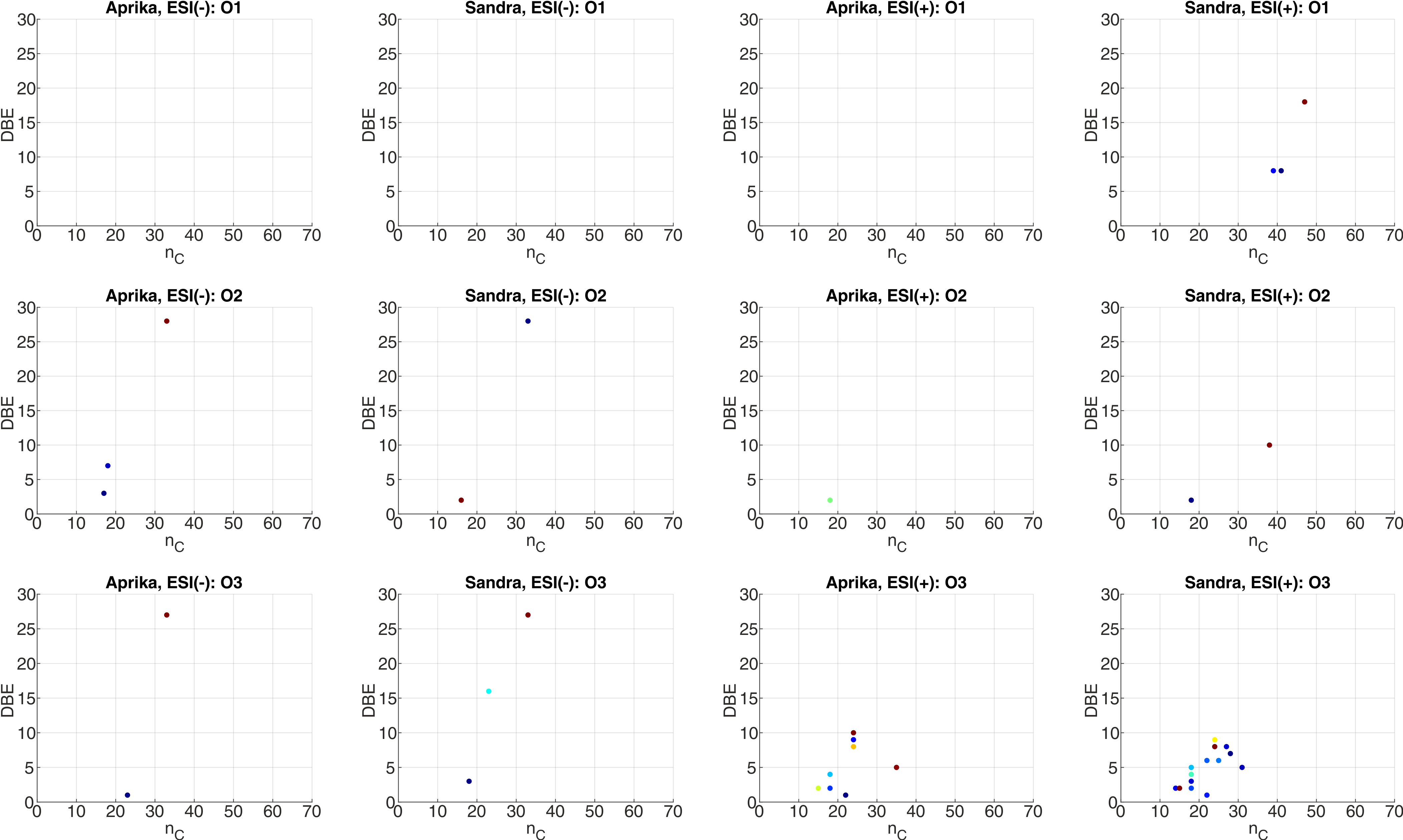

Supplement: Supplementary file 1 [file plants-12-02238-s001.zip › Figure S1.tif]

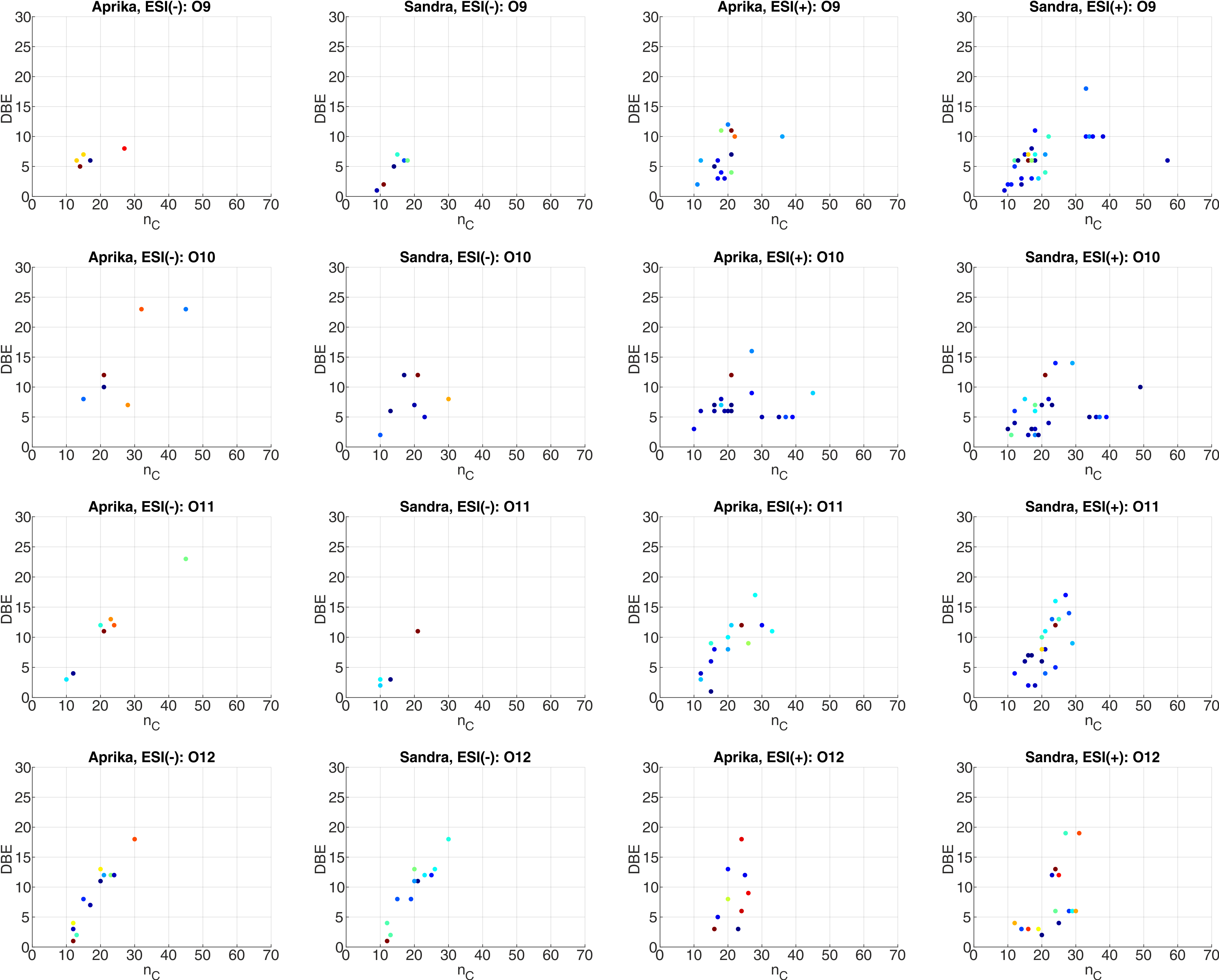

Supplement: Supplementary file 1 [file plants-12-02238-s001.zip › Figure S2.tif]

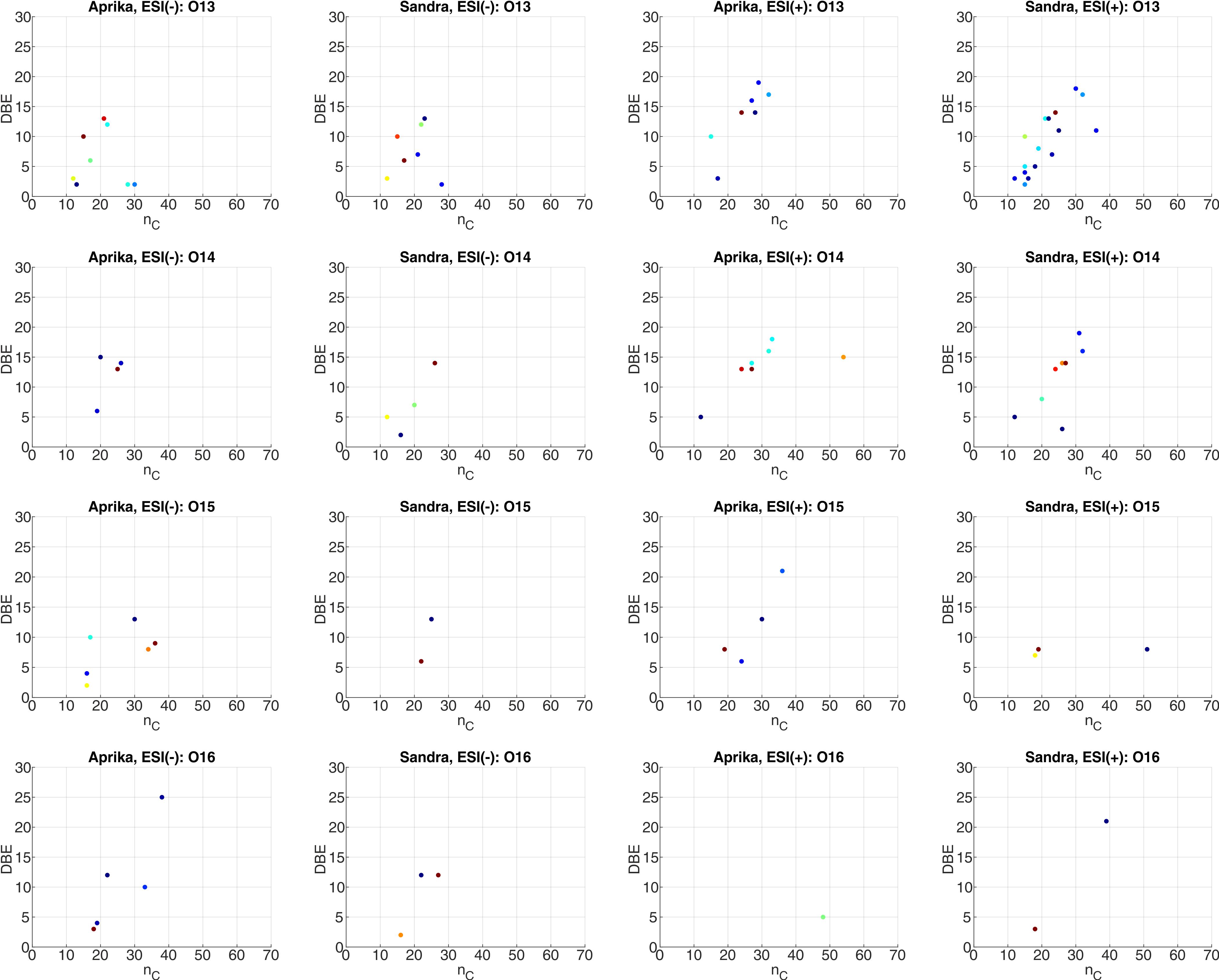

Supplement: Supplementary file 1 [file plants-12-02238-s001.zip › Figure S3.tif]

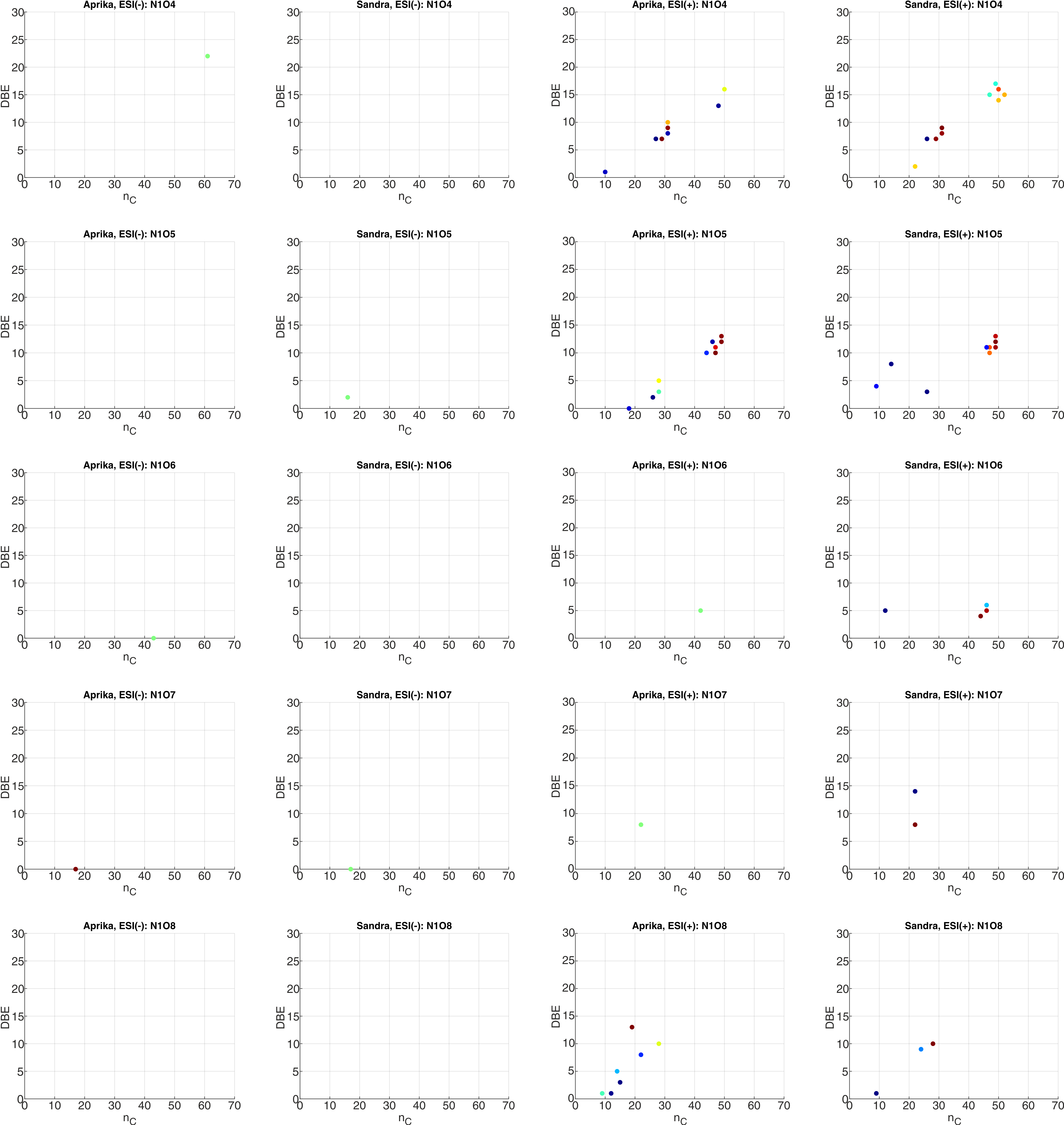

Supplement: Supplementary file 1 [file plants-12-02238-s001.zip › Figure S4.tif]

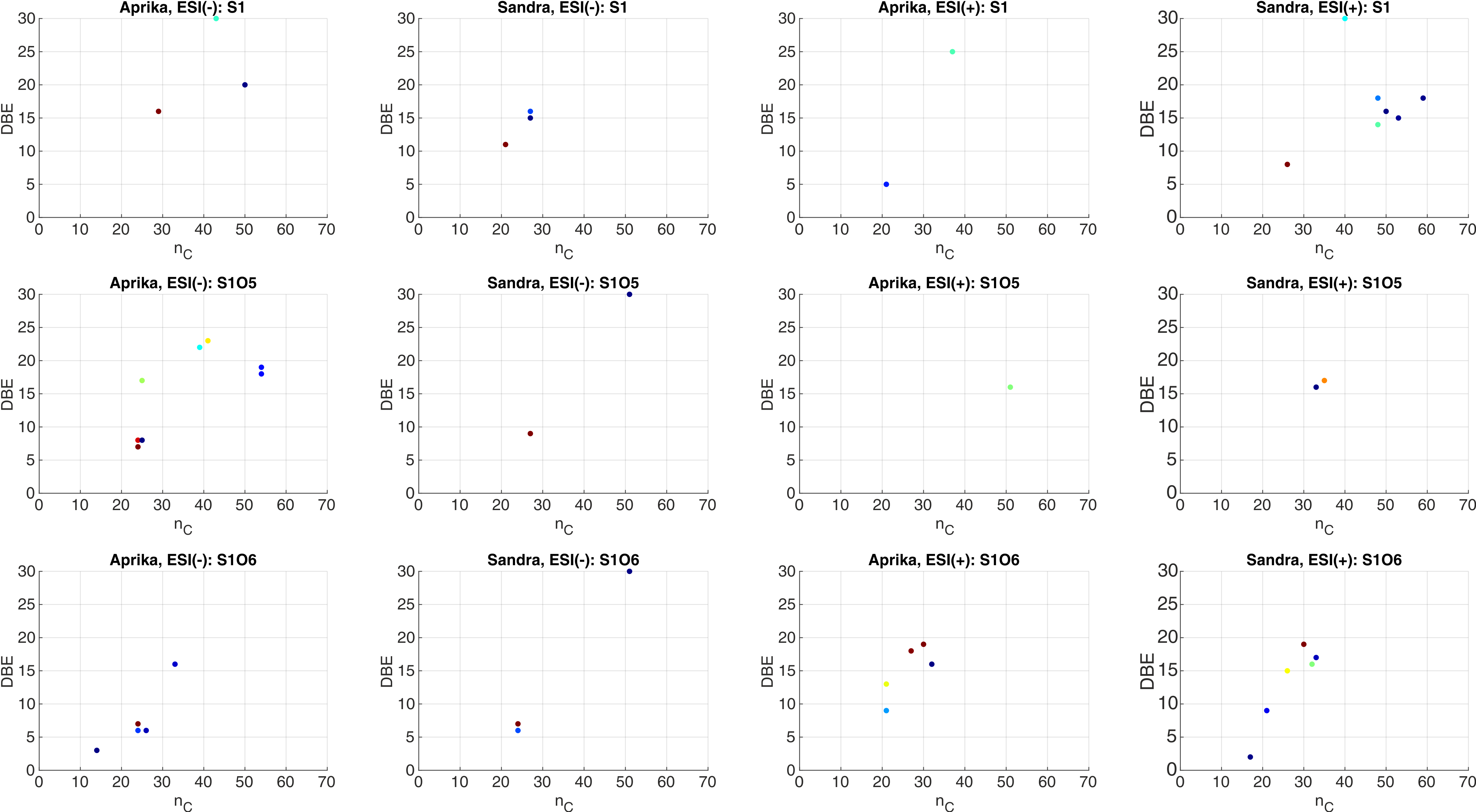

Supplement: Supplementary file 1 [file plants-12-02238-s001.zip › Figure S5.tif]
